# Reversibility of Vasalgel™ male contraceptive in a rabbit model

**DOI:** 10.1186/s12610-017-0051-1

**Published:** 2017-04-05

**Authors:** Donald Waller, David Bolick, Elaine Lissner, Christopher Premanandan, Gary Gamerman

**Affiliations:** 1 Prelabs, LLC, 33 W Chicago Ave., Oak Park, IL 60302 USA; 2 Seraphim Life Sciences Consulting, LLC, 2158 Bonaventure Drive, Suite 101, Vienna, VA 22181 USA; 3 Parsemus Foundation, PO Box 2246, Berkeley, CA 94702 USA; 4 Department of Veterinary Biosciences, College of Veterinary Medicine, The Ohio State University, 1925 Coffey Road, Columbus, OH 43215 USA

**Keywords:** Male contraception, Polymer, Reversibility, Long-acting reversible contraceptive, SMA

## Abstract

**Background:**

Development of a non-hormonal long-acting reversible contraceptive for men could have a significant impact on reducing unintended pregnancies. Vasalgel™ is a high molecular weight polymer consisting of styrene-alt-maleic acid (SMA) dissolved in dimethyl sulfoxide being developed as a reversible male contraceptive device. It forms a hydrogel when implanted into the vasa deferentia, which prevents the passage of sperm. Previous studies in the rabbit have proven its efficacy, durability and rapid onset. This study evaluates the capacity to restore sperm concentrations in ejaculates after a reversal procedure.

**Methods:**

Sodium bicarbonate was injected into the vasa deferentia after fourteen months of azoospermia following the injection of two device variations (Vasalgel 100 and Vasalgel 80). Semen samples were then collected for six months and sperm characteristics were compared to baseline levels. Samples of vasa deferentia were obtained for histological examination.

**Results:**

Spermatozoa were present in all subject ejaculates after the reversal procedure. Sperm concentration and sperm motility were similar to baseline levels after reversal, while sperm forward progression was significantly lower and normal acrosomes were not observed. Forward progression percentages increased linearly during six months of semen collection, however, normal acrosomes were not observed at the conclusion of the study. Histologically, several vasa deferentia were clear of the device and contained an intact epithelial lining. A smaller proportion of tissues contained residual test material. A secondary intraluminal inflammatory response was seen occasionally in the tissues containing residual material. There was no difference between the two device variations for studied parameters.

**Conclusions:**

Vasalgel’s prevention of sperm transport for 14 months was reversed through an intravasal injection of sodium bicarbonate. Post-reversal sperm concentrations and motility returned to baseline levels during the six-month follow up. Residual material in the vas lumen or compromised epididymal and vas deferens function may be resulting in reduced forward progression and loss of acrosomes during transit through the vas. Reduced forward progression and the lack of normal acrosomes strongly suggest impaired sperm function.

## Abstract (fr)

**Contexte:**

Le développement d’une contraception réversible, de longue durée et non hormonale pour les hommes pourrait avoir un impact significatif sur la réduction du nombre de grossesses non désirées. Le Vasalgel ™ est un polymère de haut poids moléculaire constitué d’acide styrène-alt-maléique (SMA) dissous dans du diméthylsulfoxyde, en cours de développement comme moyen réversible de contraception masculine. Une fois injecté dans les canaux déférents, il forme un hydrogel qui fait obstacle au passage des spermatozoïdes. De précédentes études chez le lapin ont prouvé son efficacité ainsi que la rapidité et la persistance de son effet. La présente étude a évalué la capacité de restauration de la concentration de spermatozoïdes dans les éjaculats obtenus après une procédure de réversion.

**Matériel et Méthodes:**

Du bicarbonate de sodium a été injecté dans chaque canal déférent après quatorze mois d’azoospermie secondaire à l’injection de deux formulations du produit testé (Vasalgel 100 et Vasalgel 80). Des échantillons de sperme ont ensuite été recueillis pendant six mois et les caractéristiques des spermatozoïdes comparées aux valeurs de départ. Des échantillons de canal déférent ont été prélevés pour analyse histologique.

**Résultats:**

Des spermatozoïdes furent retrouvés dans les éjaculats de tous les animaux après la procédure de réversion. La concentration de spermatozoïdes et le pourcentage de spermatozoïdes mobiles après réversion étaient similaires aux valeurs de départ, mais le pourcentage de spermatozoïdes à mobilité progressive directionnelle était significativement moindre que celui des valeurs de départ, et aucun acrosone normal n’a été observé. Les pourcentages de spermatozoïdes à mobilité progressive directionnelle ont augmenté linéairement durant les six mois de recueil du sperme ; cependant, aucun acrosome normal n’a été retrouvé à la fin de l’étude. Au plan histologique, de nombreux canaux déférents ne contenaient plus aucune trace du produit testé et étaient bordés par un épithélium intact. Une plus faible proportion d’échantillons contenait des traces du produit testé. On a occasionnellement observé une réaction inflammatoire secondaire dans la lumière des canaux contenant des résidus de produit testé. Il n’a été retrouvé aucune différence entre les deux formulations du produit testé pour les paramètres étudiés.

**Conclusions:**

L’obstacle au transport des spermatozoïdes pendant 14 mois réalisé par le Vasalgel™ a été supprimé par une injection intra canalaire de sodium de bicarbonate. Après la réversion, la concentration de spermatozoïdes et le pourcentage de spermatozoïdes mobiles reviennent à leur valeur de départ durant les six mois de suivi. La présence dans la lumière du canal de résidus du produit testé, ou une altération des fonctions épididymaire et déférentielle, pourraient entraîner la réduction de la mobilité progressive directionnelle et la perte des acrosome durant le transit des spermatozoïdes dans les canaux. La réduction de la mobilité progressive et l’absence d’acrosomes normaux suggèrent fortement une altération de la fonction spermatique.

**Mots-clés:**

Contraception masculine, Polymère, Réversibilité, Contraception réversible de longue durée, Acide styrène-alt-maléique (SMA)

## Background

Long-acting reversible contraception (LARC) methods are recommended for pregnancy prevention because of their potential for high effectiveness and low burden to users. LARC options available to women (IUDs and implants) have been associated with high levels of continuation [1]. While LARCs often cost more initially, they are among the most cost effective birth control methods given their high efficacy rate and duration of use, especially when considering the costs of unintended pregnancy [2, 3].

Having a variety of new fertility control options for men and women would accommodate people of different ages and life stages [4]. The availability, variety and effectiveness of LARCs for women have had a significant impact on reducing the unintended pregnancy rate [5]. It is likely that similar options for men would also have positive impacts on reproductive health. However, currently there are no LARCs for men. Vasectomy, while long-acting, is generally considered permanent due to difficulties of reversal, the expense, and lower success rate in restoring fertility [6]. Other options (condoms, withdrawal) are short-term solutions with high pregnancy rates for typical use [7].

Research on male contraception has revolved around both hormonal and nonhormonal methods that suppress sperm production, disrupt sperm maturation or function, or alter sperm transport or motility [8]. A promising advancement is Vasalgel, a high molecular weight polymer of styrene-alt-maleic acid (SMA acid) being developed as a LARC for men. The device is injected into the vas deferens to block sperm from transiting through the vas deferens. The implant remains in a soft gel-like state, with the ability to adapt to the interior lumen of the vas deferens and minimize any accommodation of the vas to the presence of the material. It forms a hydrogel which allows transit of water soluble molecules but not larger structures such as spermatozoa.

Initial research on rabbits has confirmed that Vasalgel provides a rapid onset of azoospermia with durability over a 12 month test period [9]. The study also confirmed the efficacy of the SMA acid in Vasalgel as compared to styrene-maleic anhydride (SMA anhydride), which is the basis of a similar product called RISUG that has been studied in India for over three decades [10]. Vasalgel’s composition gives it important advantages for large-scale manufacturing and product stability which should facilitate regulatory approval.

The purpose of the present study was to evaluate the efficacy of a sodium bicarbonate injection into the vasa deferentia to reverse Vasalgel-induced azoospermia in rabbits.

## Methods

### Devices

Vasalgel devices consisted of 25% solutions by weight of SMA in dimethyl sulfoxide (DMSO). The average molecular weight (Mw) of the SMA anhydride was 330 kDa based on standardized gel permeation chromatography (GPC) methodology (Jordi Labs, Mansfield, MA, USA). The SMA acid was made by hydrolysis of the anhydride and had a Mw of 360 kDa. One device contained only SMA acid and is referred to as Vasalgel 100. A second device contained a mixture of 80% SMA acid and 20% SMA anhydride by weight and is referred to as Vasalgel 80. The final devices were prepared and packaged in a nitrogen atmosphere in 4 ml glass vials by Polysciences, Inc. (Warrington, PA, USA).

### Subjects, housing and care

The study was performed using 7 mature azoospermic male New Zealand White rabbits (Harlan Laboratories, Oxford, MI) from the Vasalgel contraceptive efficacy study. The animals averaged 1.9 years of age (SD = 0.07 weeks) and weighed an average of 4.6 kg (SD = 0.49 kg) (see [9] for details of care and housing). Three mature females were used as teasers for the collection of semen. All animal procedures were approved by the Loyola University Medical Center, (Maywood, IL) Institutional Animal Care and Use Committee.

### Experimental design

Baseline semen sample collections were obtained prior to implantation of the Vasalgel device during the contraceptive efficacy study [9]. All rabbits received bilateral vas deferens implants of Vasalgel 100 (*n* = 2) or Vasalgel 80 (*n* = 5). Fourteen months (SD = 0.75 months) after implant the azoospermic males underwent a reversal procedure to remove the Vasalgel and return patency to the vasa deferentia. Semen collections began two weeks following the procedure and continued for six months to establish the presence and quality of spermatozoa.

### Implantation of devices

Rabbits were injected with the devices as previously described [9]. Briefly, the vasa deferentia of anesthetized rabbits were externalized and injected with approximately 100–120 μl of the device in about 30–40 s using a 24 gauge 1.6 cm catheter (Quik-Cath by Baxter, Deerfield, IL). The catheter was then removed, the vasa deferentia gently compressed for about 30 s and the vasal muscularis at the site of injection identified with a 6–0 Prolene suture. The vas deferens was returned to the spermatic cord and the site closed with 4–0 nylon sutures.

### Reversal procedure

Animals were weighed, administered an antibiotic (Baytril® [Bayer Healthcare, KS, USA] 5 mg/kg) and then anesthetized with an intramuscular injection of xylazine HCl (4 mg/kg) and ketamine HCl (50 mg/kg) and a subcutaneous injection of acepromazine maleate (1.0 mg/kg). A 1 cm suprapubic transverse incision was made in the midline approximately 2 cm cephalad to the pubic symphysis. The spermatic cords were brought up through the incision and isolated. The cremasteric fascia was incised in a longitudinal fashion and the vas deferens isolated with its blood supply. The suture marking the site of implantation was identified.

A 19 gauge needle was used to enter the vasa deferentia approximately 0.3 cm from the Vasalgel injection site (towards the epididymis). A two lumen 24 gauge 15 cm in length catheter was then inserted approximately 0.5 cm into the vas deferens and advanced to a maximum distance of approximately 8 cm when resistance to further insertion occurred. The catheter was then withdrawn about 1 cm and a 20 ml syringe attached to the outer lumen of the catheter to flush the vasa with sodium bicarbonate solution (2 M, pH 8.0, Hospira, Lake Forest, IL, USA). The central lumen provided a path for any off gassing due to the reaction of Vasalgel and the bicarbonate as well as any excess fluid being instilled. Gentle pressure was initially applied to the syringe until resistance was encountered, the pressure stopped for about 15–30 s and then pressure again applied. This process was repeated several times until the gentle pressure on the syringe allowed unrestricted flow of the bicarbonate through the vas. The number of repeated cycles varied between vasa and the total volume required prior to initially obtaining unrestricted flow was about 2 to 5 ml of the bicarbonate solution. A maximum of 10 ml was injected.

### Semen collection and evaluation

Semen collections were performed using a warmed artificial vagina semen-collection device designed for use with the rabbit and a “teaser” female to encourage mounting [11]. Semen specimens were evaluated for volume, total sperm count, motility and forward progression using manual methods.

### Euthanasia and necropsy

Rabbits were euthanized using standard procedures at the conclusion of the study and necropsied with particular attention to the reproductive tract.

### Histological examination

The vasa deferentia from treated animals were harvested and immersed in 10% neutral buffered formalin for fixation. The tissues were processed, sectioned and stained with hematoxylin and eosin utilizing standard methods for evaluation.

### Data analysis

Data were summarized by subject, device group (Vasalgel 100 and Vasalgel 80) and condition (baseline and post-reversal) for each sperm measurement. The reversibility of the device was evaluated by comparing the mean sperm parameters (sperm concentration, forward progression and motility) for each subject by condition and by device. Initial evaluation of the data set revealed deviations from normal distribution. Thus, nonparametric tests were applied to determine any difference between the Vasalgel 100 and Vasalgel 80 groups (Mann Whitney U test) and evaluate any difference between the measures at baseline vs. post-reversal (Wilcoxon Matched Pairs test) using Statistica (StatSoft, Inc., Tulsa, OK, USA). A significance level of *p* < 0.05 was determined. Data are presented as mean ± standard deviation.

## Results

A total of twelve vasa deferentia were successfully flushed with 2–10 ml of sodium bicarbonate solution (average 6.8 ± 4.70 ml). One vas in each of two rabbits in the Vasalgel 80 group, was not readily accessible or identifiable and could not be flushed. The flushing medium resulted in off-gassing of CO_2_ and the observation of some foam as it exited at the insertion site, flowing around the outside of the catheter. Minor distention of the vas deferens was observed during flushing in two subjects.

The average number of baseline semen samples collected prior to implantation was 2.4 ± 0.5. Baseline sperm concentration average prior to the administration of the test devices was 221 ± 46 × 10^6^ sperm/ml with 69.4 ± 10.2% motility and 40.9 ± 6.0% forward progression. Animals were azoospermic prior to the reversal procedure. Semen collection commenced 14 days after the reversal procedure and first semen samples obtained on average at 18.9 ± 5.5 days post-reversal and continued for an average of 22.6 ± 3.3 weeks. Eighty percent of attempted semen collections were successful and the number of analyzable collections was 15.9 ± 6.3 per subject during the post-reversal period.

Spermatozoa were observed in the ejaculates of all subjects at an average of 23.7 ± 9.3 days after the reversal procedure. Measurable sperm concentration was evident at the first sperm collection in four subjects (two Vasalgel 100 and two Vasalgel 80), with one as early as 12 days post-reversal. Sperm concentration was measurable in the other three rabbits after several additional collections. Sperm concentrations were initially higher in the first collections after the reversal procedure in several animals and also higher compared to the baseline (average 301.9 ± 57.2 × 10^6^ sperm/ml). Motility averaged 62.3 ± 9.6% after reversal. Forward progression averaged 5.3 ± 0.9% after reversal, increasing from less than 3% for all subjects immediately after reversal to more than 11% for all subjects at the last sample.

**Fig. 1:**
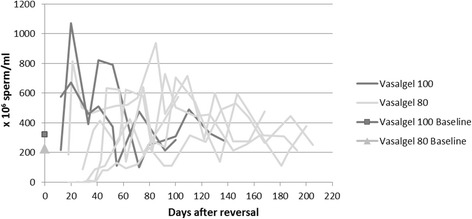
Sperm concentration (x 10^6^) following reversal for the seven rabbits showing the baseline average per group and trends for the two different devices. No significant difference between the devices was found

As illustrated in Fig. 1, some of the subjects in the Vasalgel 80 group had low initial sperm concentrations, but this observation is difficult to interpret due to high variation across individuals and over time as well as the small number of subjects. Two of the Vasalgel 80 subjects only had one vas deferens flushed. Of these, one had sperm present in the first semen sample and the other had sperm by the fifth sample. Statistically, the three sperm parameters (concentration, motility and forward progression) did not differ for the Vasalgel 100 vs. Vasalgel 80 (all *p* > 0.05). Therefore, all groups were combined for further statistical tests.

**Fig. 2:**
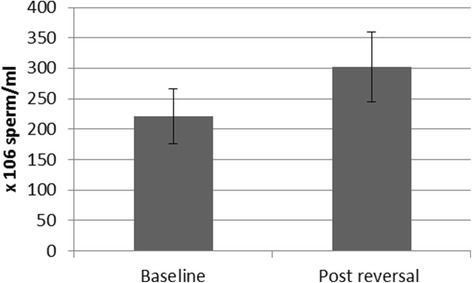
Sperm concentration (x 10^6^) during baseline and following reversal procedures. Sperm concentration returned to baseline levels following reversal. No significant difference between baseline and post-reversal

**Fig. 3:**
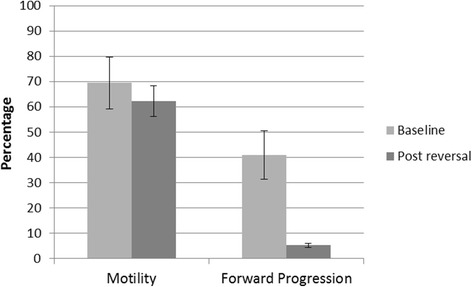
Sperm motility and forward progression percentage at baseline and following reversal procedures. Average motility returned to baseline levels following device reversal. Forward progression was significantly lower following device reversal (*p* < 0.02)

**Fig. 4:**
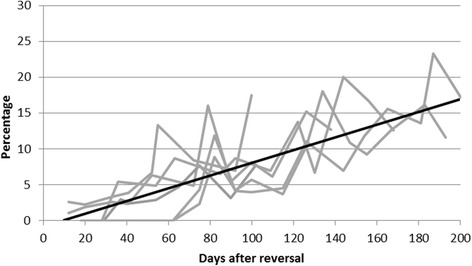
The percent of sperm showing forward progression after the device reversal for each rabbit. A linear trend line (y = 0.089x − 0.798, *R* ^2^ = 0.90) shows that the forward progression measures are increasing over time

Statistical comparisons between the baseline sperm measurements and those obtained after the reversal procedure were conducted to determine whether the values had returned to normal. Sperm concentration was not significantly different between baseline and post-reversal (Wilcoxon Z = 1.01, *p* < 0.31) (see Fig. 2). Sperm motility was also not significantly different when comparing baseline to post-reversal (Wilcoxon Z = 1.25, *p* < 0.17) (Fig. 3). Forward progression was significantly lower after the reversal procedure (Wilcoxon Z = 2.37, *p* < 0.018) (Fig. 3) with a positive trendline (*R* ^2^ = 0.90 (see Fig. 4)).

The normal large rabbit acrosome was not observed on spermatozoa during post reversal semen analysis. This condition was observed throughout the period of time in which sperm were obtained and analyzed post reversal.

Gross observations of the reproductive tract during necropsy indicated that the testes were normal in size and color. The prostate and epididymides appeared normal except one subject had a darkened epididymis on the right side and two subjects had a smaller than normal epididymis. Other gross observations of the vasa deferentia included adhesions, swelling or distention at the end near the epididymis (5/12) and thinning at the prostate end (8/12).

**Fig. 5:**
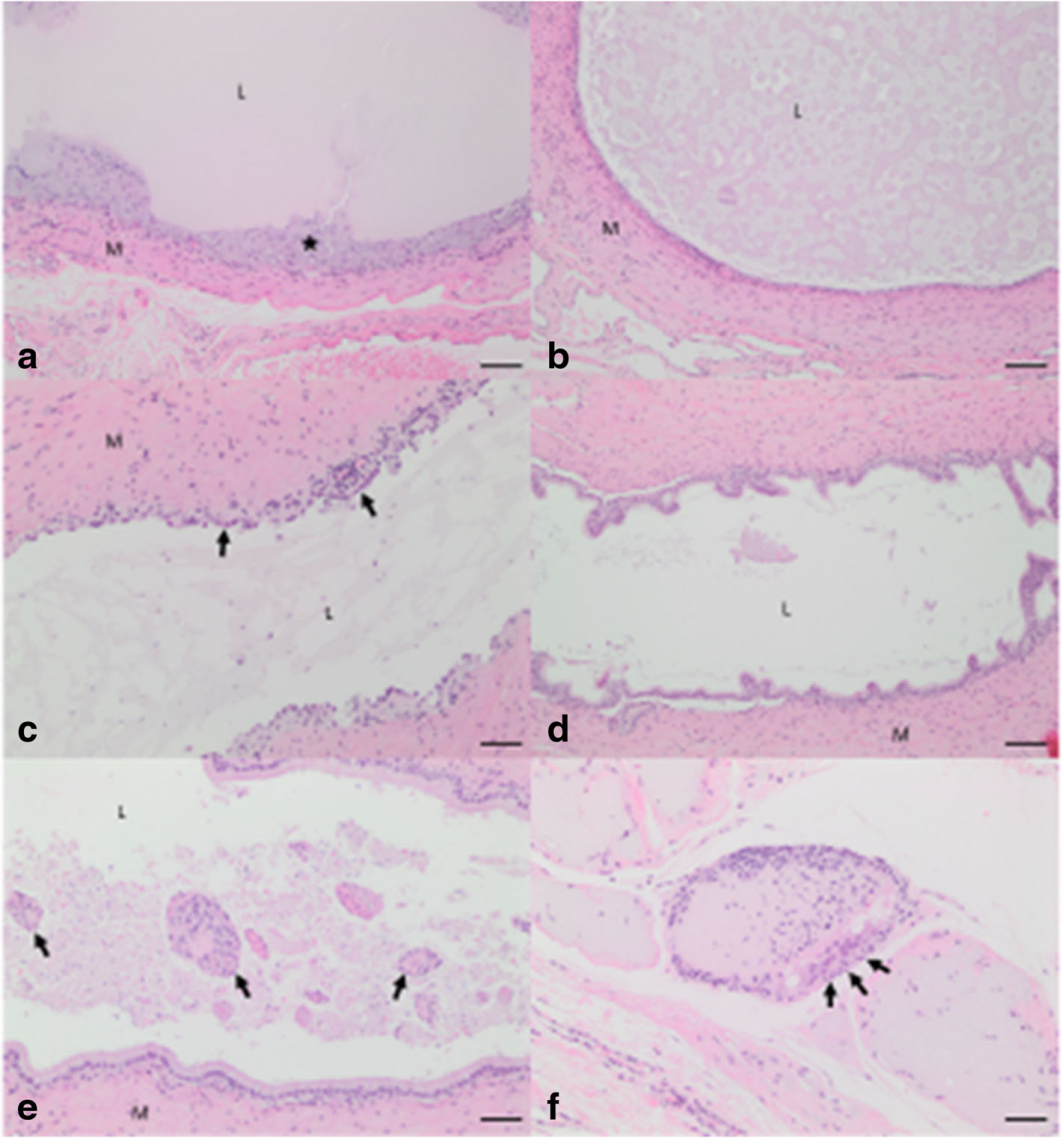
Rabbit vas deferens examined after device reversal procedure. **a** Longitudinal section (100X magnification) of vas deferens, containing residual material appearing as homogenous luminal substance. The ★ depicts a layer of granulomatous inflammation which replaces the mucosal epithelial cells. Muscularis (M), vas deferens lumen (L). Bar = 100 μm. **b** Cross section (100X magnification) of vas deferens containing fragmented residual material. Muscularis (M), vas deferens lumen (L). Bar = 100 μm. **c** Longitudinal section (200X magnification) of vas deferens. Fragmented material is present in the lumen. The mucosal epithelium is attenuated in this photomicrograph (*arrow*). Muscularis (M), vas deferens lumen (L). Bar = 50 μm. **d** Longitudinal section (100X magnification) of vas deferens. The lumen is empty and tall columnar epithelium is present. Muscularis (M), vas deferens lumen (L). Bar = 100 μm. **e** Additional longitudinal section (200X magnification) of vas deferens. Clumps of spermatozoa are present in this image (arrows). Muscularis (M), vas deferens lumen (L). Bar = 50 μm. **f** Extraluminal adventital material with associated granulomatous inflammation (200X magnification). Arrows depict a multinucleated giant cells adjacent eosinophilic to amphophilic material in the interstitium. Bar = 50 μm

Twenty segments of vas deferens from five of the rabbits were examined (two segments from the treated left and right vas deferens from each animal). The vasa deferentia in the animals exhibited a variety of intraluminal changes (See Fig. 5a–f). Hydrogel residue was observed in 4 of 20 segments (See Fig. 5a, b and c). Two of these segments were from one animal with residue observed in segments from both vasa deferentia. The material appeared as a non-fibrillar homogenous amphophilic material. Frequently, clear fracturing was observed in this material and was interpreted as a processing artifact. In these animals, the mucosal epithelium was variably replaced by round to polygonal cells with abundant eosinophilic cytoplasm and round to oval nuclei. These cells often exhibited close association to each other and were interpreted as epithelioid macrophages. Similar material present in the lumen of the vas deferens of these animals was also observed in the surrounding interstitial connective tissue and interpreted as extraluminal hydrogel. The accumulation of this material was often arranged in a multifocal to coalescing nodular pattern and was associated with a surrounding rim of epithelioid macrophages and multinucleated giant cells (See Fig. 5f). This was a similar change to that seen in the non-reversed animals in the previous study [9].

In seven of the 20 segments, intraluminal fragmented material was observed with similar staining properties as the material seen in the intact device plugs. Some of these fragmented hydrogel regions contained scattered spermatozoa (See Fig. 5e). Nine of 20 segments exhibited moderate epithelial attenuation and flattening characterized by a cuboidal to squamous appearance. Segmental mucosal epithelial loss without inflammation was observed in some regions in this group. This epithelial change was not always associated with the presence of an intact plug or fragments of intraluminal device (i.e. was seen in segments of clear vas deferens). The remaining segments (7 of 20) exhibited intact epithelium that appeared columnar and frequently had a ciliated apical surface (See Fig. 5d).

## Discussion

The historical lack of focus on male contraceptive development entails cultural, financial and technical aspects [12]. Contraceptives have been aimed at women, who bear the responsibility for child-bearing and also traditionally for child-rearing. With societal shifts in gender roles and with modern paternity testing, men have taken on more parenting responsibility. Questions of trust in male partners to take birth control also continue to play into attitudes about the importance of developing male contraceptives, yet many commenters are not aware that as much as a quarter of the world’s population already relies on modern or traditional male methods. These changes are reflected in recent studies and surveys indicating that men are not only willing to share responsibility for contraception but demand greater reproductive control [8, 12]. Even with increased demand, male contraceptive development has still been challenged by liability issues, politics, religion and lack of public/private investment [4, 13]. While research has explored a number of different male contraceptive options, no commercially available LARCs for men are on the market.

A male LARC has the potential to decrease the rate of unintended pregnancies, provide men with additional options to control their reproduction, and offer couples an alternative when female contraceptive methods are problematic. Vasalgel is a promising male LARC, and studies in rabbits prove that Vasalgel not only provides rapid and durable contraception [9], but also a return of spermatozoa in the ejaculate shortly after undergoing a reversal procedure with a sodium bicarbonate solution.

In the current study the vasa deferentia of Vasalgel-induced azoospermic rabbits were flushed with a sodium bicarbonate solution to reestablish patency for the transport of spermatozoa. Bicarbonate solutions have been used in previous studies to reverse a similar hydrogel in the rat model [14, 15]. DMSO was not considered for dissolving the hydrogel plug since it could cause increased inflammation during prolonged contact with the vas deferens [16].

Spermatozoa concentrations returned to normal after the reversal procedure. The initially higher sperm concentrations were probably due to increased epididymal sperm reserves resulting from long term blockage of sperm transit [17, 18]. Motility also returned to baseline levels following reversal, but forward progression was significantly lower in the post-reversal period. A trend of increasing forward progression values over time indicated that a slow recovery process was occurring.

The normal time for spermatogenesis and transit to epididymal reserves is about 40 days [19]. Transit time from the testes through the epididymis is about 10 days [19, 20]. Thus, the post reversal semen collections were performed after a minimum of 2.5 cycles of sperm production to ensure newly produced spermatozoa were being ejaculated.

A lack of normal acrosomes on the spermatozoa was observed throughout the post reversal semen evaluations. Previous studies of other hydrogels indicate residual material in the vas lumen may also affect the spermatozoa during transit down the vas [21–23]. The decrease in normal forward progression and lack of normal acrosomes may indicate compromised epididymal and/or vasal support of sperm maturation and transport which can lead to reduced or complete infertility. Although the reversal process successfully re-established the transport of spermatozoa into the ejaculate, the ejaculated spermatozoa have not returned to normal.

The impact of the post-reversal sperm characteristics on pregnancy rates is unknown in this study since mating with females was not included in the study protocol. Previous work in artificial insemination in rabbits indicated that about 10 million motile sperm per sample were required for pregnancy [24]. In our study, rabbits averaged 187 million motile sperm with 16.0 million sperm showing forward progression. We would expect mating success based solely on sperm concentration and motility. However, the reduced forward progression and the lack of a normal acrosome would negatively impact fertility. Normal forward progression and acrosomes are important predictors of fertility. The forward progression recovery trend provides evidence that the return to completely normal spermatozoa may occur over a longer period of time and restore fertility.

The contact of spermatozoa with residual material during transit through the vas deferens may have negatively impacted morphology and functionality of the spermatozoa as observed in previous studies with a similar material [21–23]. Fertility in man would most likely require a return of all semen parameters to within normal ranges.

The vasal epithelium appeared to be returning to normal following the reversal procedure. Although some areas were observed with epithelial attenuation and flattening, most of the vasal epithelium appeared to have an intact structure with normal columnar appearance and a ciliated apical surface.

Nonetheless, all animals had spermatozoa present in the semen following the reversal procedure to confirm the reestablishment of patency in the vasa deferentia.

## Conclusions

The blockage of sperm transit for 14 months following Vasalgel implantation was rapidly reversed through an intravasal injection of sodium bicarbonate. Sperm counts returned to normal in all of the subjects over the period of observation. Post-reversal concentration of sperm and sperm motility percent returned to baseline levels during the six-month follow up, with the reduced sperm forward progression exhibiting a recovering trend. Normal acrosomes were not observed over the post reversal evaluation period. Further investigations are needed to determine if a longer period of time would allow complete recovery of the vasal structures or if improved flushing procedures are needed to completely remove the Vasalgel leading to the return of normal sperm morphology and forward progression.

Study results support continued development of Vasalgel as a non-hormonal LARC for men.

## Abbreviations

DMSO: Dimethyl sulfoxide
GPC: Gel permeation chromatography
Mw: Molecular weight
SD: Standard deviation
SMA acid: Styrene maleic acid
SMA anhydride: Styrene maleic anhydride
